# From access to sustainability: understanding telemedicine-based buprenorphine induction through a RE-AIM lens

**DOI:** 10.1186/s13722-026-00670-6

**Published:** 2026-05-26

**Authors:** Abhishek Ghosh, Harpreet Singh Dhillon, Blessy B. George, Kashish Ranchen, Pragyapti Malav, Shalini S. Naik, B. N. Subodh, Debasish Basu

**Affiliations:** Drug Deaddiction and Treatment Center, Department of Psychiatry, Postgraduate Institute of Medical Education and Research, Chandigarh, 160012 India

**Keywords:** Telemedicine, Opioid agonist maintenance treatment, Qualitative, Interviews, India

## Abstract

**Background:**

Telemedicine-Assisted Buprenorphine Induction (TABI) is a hybrid treatment model combining in-person initiation with remote follow-up, developed to improve access to opioid agonist maintenance treatment (OAMT) in India. While clinical trials demonstrated its non-inferiority to standard care, process evaluations are essential to understand contextual factors influencing its implementation and sustainability.

**Methods:**

A qualitative process evaluation, guided by the RE-AIM framework, was conducted alongside a randomized controlled trial comparing TABI with in-person induction. Fourteen patients with opioid use disorder (OUD) who underwent TABI in the previous three months and four TABI providers were interviewed using semi-structured guides. Thematic analysis was conducted iteratively, and data saturation was confirmed at the 14th patient interview. Data were analyzed using the framework method, combining deductive coding based on RE-AIM domains.

**Results:**

TABI improved treatment reach by reducing travel and time barriers, although digital literacy challenges, network instability, and providers’ bias for judging clinical suitability might constrain implementation. Participants reported positive outcomes, including reduced opioid use and functional improvements. Adoption was supported by family engagement and provider trust but hindered by stigma and technological barriers. Implementation fidelity was largely maintained, with providers adapting workflows to address connectivity disruptions. Sustainability was linked to structured follow-up, hybrid care reinforcement, and motivational interventions.

**Conclusion:**

TABI is an acceptable, feasible, and effective model for OAMT delivery in low-resource settings. Future scale-up should address digital inequities, strengthen psychosocial and motivational supports, embed periodic in-person reinforcement, and integrate stigma-reduction strategies to optimize long-term treatment outcomes.

**Clinical trial number:**

Not applicable.

**Supplementary Information:**

The online version contains supplementary material available at 10.1186/s13722-026-00670-6.

## Introduction

Opioid use disorder (OUD) continues to pose a major global public health burden, with over 40 million individuals estimated to be dependent on opioids and nearly half a million deaths annually attributed to opioid-related harms [1]. Opioid agonist maintenance treatment (OAMT), particularly with buprenorphine, has been widely recognized as a cornerstone in OUD management due to its efficacy in reducing withdrawal symptoms, opioid cravings, and overdose mortality [2]. Buprenorphine is a high-affinity, partial agonist at the µ-opioid receptor with slow dissociation, producing effective suppression of withdrawal and craving while conferring a ceiling effect on respiratory depression that improves safety relative to full agonists. Its pharmacology allows flexible dosing and reduces overdose risk, though high receptor affinity can precipitate withdrawal if initiated too soon after full-agonist use. Buprenorphine may be co-formulated with naloxone to deter parenteral misuse without diminishing therapeutic effects when taken sublingually. These properties, together with acceptable tolerability and suitability for hybrid or take-home models, make buprenorphine particularly well-suited to expanding access to OAMT in resource-constrained settings. However, access to evidence-based OUD treatment remains limited, particularly in low-resource settings where geographic, logistical, and stigma-related barriers hinder care engagement [3, 4]. These challenges highlight the need for scalable, patient-centered models of care.

In India, despite the inclusion of buprenorphine on the essential medicines list and the growing expansion of addiction services, the uptake of OAMT is undermined by systemic constraints such as clinic-based supervision requirements, a lack of trained providers, and legal regulations under the Narcotic Drugs and Psychotropic Substances (NDPS) Act [5]. Emerging global evidence underscores that barriers to OAMT are not merely limited to regulatory or supply-side constraints but also include complex process-level challenges, such as rigid programmatic rules, inconvenient dosing schedules, and limited take-home options, that reduce flexibility and deter sustained engagement [6]. Social stigma, both from the community and within familial or cultural settings, further marginalizes individuals seeking care. Moreover, a lack of integration between primary care systems and specialized addiction services continues to fragment the continuum of care [6].

Telemedicine has emerged as a transformative approach to delivering health services, particularly during the COVID-19 pandemic, demonstrating the feasibility of remote management of chronic conditions, including OUD [7]. Telemedicine-delivered medications for opioid use disorder (TMOUD) rapidly expanded during the pandemic in response to regulatory flexibilities and the urgent need to maintain treatment continuity. This shift was not merely technological but structural, enabling services to bypass longstanding barriers such as prescriber shortages, stigma, limited clinic hours, and geographic distance [8]. Various hybrid models, ranging from hub-and-spoke to street-based delivery, emerged across diverse settings, including syringe exchange programs, prisons, and rural communities [8]. These models successfully leveraged telecommunication infrastructure to extend the reach of care and personalize patient engagement while maintaining treatment fidelity and retention rates comparable to in-person services [8]. In India, the Telemedicine Practice Guidelines (2020) [9] established the regulatory framework for remote care at the start of the pandemic and remain in effect. However, MOUD provision is additionally regulated under NDPS and related rules; accordingly, TABI employed telemedicine for assessment, counseling, and structured follow-up, while initiation and dispensing were conducted in person in line with prevailing regulations.

Telemedicine-assisted buprenorphine induction (TABI), a hybrid model involving in-person initiation followed by remote follow-up, was recently evaluated in a randomized controlled trial in India. The trial found TABI to be non-inferior to standard in-person induction in terms of treatment retention and clinical outcomes [10]. Our findings build upon prior feasibility research in India, demonstrating that patients found TABI acceptable and providers found it logistically feasible within an outpatient care setting [11]. Yet efficacy and feasibility do not resolve implementation: who is reached or left out, how fidelity holds under unstable connectivity, how refills align with teleconsultations, how stigma, family dynamics, and digital literacy shape engagement, and whether the model is sustainable and equitable beyond the trial. These questions warrant a real-world process evaluation.

Process evaluations play a critical role in understanding not only whether an intervention works, but also how and why it works [12]. The RE-AIM framework (Reach, Effectiveness, Adoption, Implementation, Maintenance) offers a structured lens to assess both individual- and system-level dynamics that influence the success and scalability of complex interventions such as TABI [13]. By synthesizing qualitative insights from patients and providers, this process evaluation aims to unpack the mechanisms, contextual facilitators, and barriers shaping the delivery of telemedicine-based OAMT in India. Findings from this evaluation can inform future adaptations of the model and guide health policy reform aimed at improving access to addiction care.

## Methods

### Study design and theoretical framework

We conducted a qualitative process evaluation embedded within a randomized controlled trial that compared TABI to standard in-person care. The process evaluation was guided by the RE-AIM framework (Reach, Efficacy/Effectiveness, Adoption, Implementation, and Maintenance) to systematically examine the contextual and implementation factors shaping intervention delivery and outcomes. The use of the framework method facilitated a structured approach to analyzing qualitative data and supported comparison across cases and groups. The Consolidated Criteria for Reporting Qualitative Research (COREQ) were followed to ensure comprehensive reporting.

### Research team and reflexivity

The interviews were conducted by a trained research team comprising clinicians and social scientists from psychiatric social work and public health backgrounds; two of them had experience in delivering addiction treatment. All three had expertise with qualitative methodologies. Interviewers (PM, BBG) were not involved in clinical care provision. Nevertheless, we recognize that researcher–participant dynamics can still introduce social desirability and evaluation apprehension. To enhance credibility and trustworthiness, interviewers (public health/psychiatric social work backgrounds) received training in neutral probing and non-evaluative stance; interviews were conducted in private settings with explicit assurances that participation would not affect care. Credibility was further supported through iterative piloting and refinement of the codes and dual independent coding with consensus meetings. Reflexivity was maintained through team discussions and written and oral discussions of results, acknowledging the influence of the researchers’ professional backgrounds on data interpretation.

### Participants and setting

Participants were recruited from the main trial sites and included two groups:

Patients: A minimum of 12 participants from the TABI arm were targeted, with sampling continued until thematic saturation was achieved [14]. We recruited a purposive sample from the TABI arm of the parent trial. Non-treating research staff screened the trial roster at the end of the trial to identify potentially eligible patients and contacted them by telephone to describe the sub-study, provide an information sheet (Hindi/English), and schedule interviews with those who expressed interest.

Treatment Providers: All four providers involved in the delivery of TABI were interviewed to capture the full spectrum of provider experiences.

All interviews were conducted in private settings at a time convenient to participants. Interviews with patients were conducted in either English or Hindi, depending on the participant’s preference.

TABI is a hybrid care model designed to initiate and maintain opioid agonist treatment while minimizing in-person visits. In TABI, patients underwent an in-person clinical assessment and initiation of buprenorphine (naloxone) under supervision at the outpatient department. Buprenorphine is a controlled substance in India and cannot be initiated via telemedicine [11]. Subsequent consultations were conducted remotely via telephone or video, typically for three consecutive days and thereafter, based on clinical need, till the seventh day. In-person visits were scheduled between the seventh and 10th day [11].

The randomized non-inferiority trial which enrolled 138 participants, was conducted in outpatient addiction services within tertiary-care teaching hospital setting. Adults with opioid use disorder meeting diagnostic criteria were randomized to telemedicine-assisted TABI or standard in-person induction (first one week), with subsequent in-person stabilization and maintenance in both arms [9]. The trial cohort was predominantly male; only two women were enrolled. The present qualitative process evaluation sampled from the TABI arm and was conducted 1–6 months post-induction. Therefore, all interviewed patients had exposure to both telemedicine and in-person OAMT. Accordingly, comparative statements in this paper reflect within-participant reflections on their experiences of the two care modes rather than between-arm comparisons.

### Data collection

We used semi-structured in-depth interviews (IDIs) with topic guides developed around the RE-AIM framework. Separate guides were developed for patients and providers (see Supplementary 1–2). Interviews explored themes such as access, treatment experience, fidelity to the protocol, and perceived sustainability. Each interview lasted approximately 30–60 min. Written informed consent was obtained from all participants. All interviewed were audio-recorded.

### Data analysis

All interviews were transcribed verbatim and analyzed using the Framework Method, a systematic and flexible approach well-suited for multidisciplinary health research [15]. This method involved five key stages: familiarization with the transcripts, development of an initial thematic framework, systematic indexing (coding) of data, charting data into a framework matrix, and mapping and interpretation to generate explanatory themes.

To ensure rigor, two researchers (HSD, KR) independently read the transcripts multiple times to gain familiarity with the content. An initial codebook was developed deductively from the RE-AIM framework domains—Reach, Effectiveness, Adoption, Implementation, and Maintenance—while also allowing for inductive, data-driven codes to emerge from participant narratives. Both researchers coded the same initial transcripts independently in ATLAS.ti and met regularly to discuss coding discrepancies, refining the codebook through consensus. This iterative approach continued throughout the coding of all transcripts to maintain consistency and accommodate new insights as they arose.

Analysis proceeded concurrently with data collection using an iterative approach in which transcripts were reviewed and coded in batches to assess emerging patterns and themes. A saturation grid was employed to systematically document the emergence of new codes and to determine when thematic saturation had been achieved [14]. This grid tracked the appearance of novel themes across successive interviews, allowing the team to make informed decisions about continuing or concluding recruitment once no new codes emerged. This approach aligns with best practices in qualitative research for monitoring thematic sufficiency and ensuring analytic transparency.

The RE-AIM domains were operationalized in ways tailored to our qualitative evaluation. “Reach” was used to capture participants’ subjective perceptions of access and ease of engaging with buprenorphine treatment through TABI, rather than measuring numeric coverage. “Effectiveness” referred to perceived clinical improvements, challenges in coping, and overall treatment outcomes as described by participants. “Adoption” was defined as the facilitators and barriers influencing both patients’ and providers’ willingness to use and support TABI, including social, logistical, and motivational factors. “Implementation” described how core components of TABI were delivered in practice, encompassing fidelity to protocols, technological adaptations, and challenges encountered during delivery. “Maintenance” focused on views about sustaining treatment engagement over time and the long-term feasibility of TABI, including considerations for hybrid care models and structured follow-up.

Themes that did not clearly align with these predefined RE-AIM categories were flagged and discussed with the senior author (AG). Through team consensus, these themes were either re-integrated into the existing framework domains or reported as additional contextual insights. This structured yet flexible approach ensured that our analysis maintained deductive alignment with the RE-AIM framework while also accommodating inductive exploration of novel findings relevant to TABI implementation in this setting.

Finally, to further synthesize the qualitative findings beyond the structured RE-AIM framework, we developed an integrative conceptual model, presented as the “Mechanism of Implementation and Effectiveness Pathway.” This model was designed to illustrate the layered psychosocial and structural context in which TABI operates, mapping the patient journey from initial engagement to maintenance of treatment. Its development was grounded in the coded data, with themes systematically identified across RE-AIM domains. During analysis, the research team held iterative discussions to explore how these thematically coded elements interacted along the continuum of care. The model was intended to integrate and visualize these interconnected influences, providing an explanatory framework for understanding how TABI participants and providers perceived barriers, facilitators, and outcomes beyond the individual RE-AIM categories.

### Ethical considerations

The study was approved by the institutional ethics committee (Intramural Ethics Committee, Postgraduate Institute of Medical Education and Research, Chandigarh) overseeing the main trial. Participants received information sheets and signed informed consent forms before participation. All data were anonymized, and confidentiality was maintained throughout the study.

## Results

We interviewed 14 participants with OUD, who had undergone TABI in the last 1–6 months, and four TABI providers. All participants with OUD were men, and among the providers, two were women. The mean age was 31 years (standard deviation-SD-6.02), and the mean duration of opioid use was 7.8 years (SD 4.2). Half of the participants with OUD were educated up to high school, and three of them were illiterate. Five (35.7%) of them were from rural localities. All providers were specialized in psychiatry and had a mean clinical experience of six years.

### Framework analysis: RE-AIM

#### Reach

Persistent barriers limited the reach for some patients. Providers reported *phone access issues*, *communication barriers*, and network instability: *“At times the phone was switched off*,* numbers were invalid*,* or the video quality was so poor that we had to disconnect the video and continue with audio”* (Provider 3). Connectivity challenges were particularly acute among patients from hilly and rural areas. Participants’ initial engagement was also affected by varying levels of *technology literacy*. Some providers observed that handling devices and understanding teleconsultation processes were initially difficult for many patients, requiring detailed guidance: *“Each and everything had to be explained in detail to them.”* (Provider 1) Although most participants owned or had access to a smartphone, this did not always translate into telemedicine readiness. Some were unfamiliar with making or receiving video calls, required help from family members to connect at the scheduled time, or found it difficult to position the phone to enable effective face-to-face interaction. Thus, the barrier was less the absence of technology itself and more limited familiarity with using smartphones for clinical communication.

Despite these technological barriers, many participants still viewed TABI as improving engagement with care because it reduced the practical burden of treatment access. One patient noted, ‘It felt better not having to travel. I could talk to the doctor even when I wasn’t feeling well enough to come.’ Providers echoed this view, describing the intervention as ‘very promising’ because ‘the accessibility was better… it was way more convenient to patient and also therapist in reaching the treatment related goals without much hassle and barrier.’ These accounts suggest that, although connectivity and digital literacy posed challenges, remote contact still made treatment more reachable and manageable for many participants. This experiential pattern is consistent with the parent trial’s retention findings [9], though here we report participants’ perceptions rather than between-arm estimates.

#### Efficacy/effectiveness

Many patients reported meaningful clinical improvements after engaging with TABI. Reductions in opioid use, improvements in daily routines, and emotional stabilization were commonly reported. One patient reflected, ‘I am very happy, Ma’am. It feels like I have been given a new life after quitting addiction.’ (Patient 14, 23 years) Although this quote reflects recovery more broadly than telemedicine specifically, it captures the strength of perceived improvement reported by several participants. Providers similarly viewed TABI as effective in helping patients reach treatment goals despite practical barriers.

Many providers corroborated these outcomes, describing the intervention as “quite effective in overcoming the barriers faced in traditional hospital settings.” One provider noted, *“Effectiveness was good in reaching treatment goals*,* though I would rate it around 6 out of 10 overall*,* considering some challenges.”* (Provider 3).

However, a few patients and providers acknowledged relapse incidents and coping difficulties. One provider recounted, *“A patient missed his scheduled video calls and later relapsed because we couldn’t supervise his medication intake properly.”* (Provider 3) Patients described emotional lability early in recovery, “*I would get angry*,* and if it was affecting me on one side*,* I couldn’t bear it*”, and noted that “*on the first day*,* there was some difficulty*,” (Patient 14, 23 years) underscoring variable affect and adjustment during initiation.

External stressors were prominent and practical. Participants described ongoing difficulty obtaining leave from work and managing travel demands, noting, ‘I don’t get leave after a week because coming here takes time,’ (Patient 6, 21 years) and ‘I live alone… it’s a bit difficult to get leave to come here.’ (Patient 3, 35 years) Some also pointed out that although TABI reduced the burden of repeated in-person consultations, it did not eliminate the need to attend for medication collection and scheduled follow-up. As one patient explained, ‘the dates for the follow-up and the medicine collection overlap,’ suggesting that telemedicine eased some access barriers but did not fully resolve the structural challenges affecting continuity of care.

A few patients reported medication side effects such as fatigue and dizziness, with some providers emphasizing proactive communication about side effects during teleconsultations.

Overall, both groups found that TABI maintained the clinical effectiveness of in-person induction while offering flexibility and patient-centeredness. However, patients largely viewed effectiveness through *personal recovery experience and* expressed concerns about persistent structural barriers, while providers were concerned with *technical supervision gaps* and *clinical monitoring limitations*.

#### Adoption

Patients’ adoption of TABI was strongly influenced by the convenience and flexibility of telemedicine. Many valued that treatment could continue without frequent, costly, or logistically complex clinic visits. Providers observed that *telehealth acceptance was generally high*, especially among patients with work or family responsibilities: *“Patients accepted tele-based interventions more readily*,* saving time and travel costs.”*

Adoption was strongly shaped by close others. Patients described practical and motivational family support, “*My father comes sometimes with me*,* my mother also comes sometimes*,” (Patient 13, 25 years) and “*The doctor provided treatment*,* and my family also helped by guiding me. That’s why these changes happened*” (Patient 5, 38 years). Families often initiated contact with services: “*Someone had referred my mother… there is a drug de-addiction center at PGI… After counseling*,* we learned about it*.” (Patient 8, 32 years) Peer influence also catalyzed uptake, “*My friend was taking medication here too*,*”* (Patient 12, 41 years) *and “it was my friend… his treatment was working here… he said that I should go… Initially*,* I was not interested… My friend explained it to me well*,* and I realized that I need to stop the addiction*.” (Patient 14, 23 years).

Nevertheless, stigma persisted as a barrier to adoption for some. Some patients reported that being labeled “an addict” by family members affected their emotional readiness to commit fully to treatment. Many providers acknowledged that addressing *psychosocial vulnerabilities* early could enhance telemedicine engagement.

Decisions to enroll in TABI might be influenced by clinical assessments of patient motivation and logistical feasibility. Providers emphasized clinical suitability for tele-induction, noting that patients with stable phone access, basic digital readiness, early motivation, and a family member able to supervise at home were more likely to succeed in the remote phase, *“It worked well for patients with good family support and high motivation*,* but was less successful for those with low readiness.”* (Provider 1) Although such attributes matter for in-person MOUD as well, clinicians framed them as especially consequential for telemedicine because observation is indirect and connectivity can interrupt real-time monitoring. Providers described informal checks for device/number stability, basic video comfort, and a helper to supervise early dosing: “*the phone used to be switched off… the number… was invalid… the video quality was so poor that we had to disconnect the video and continue the audio… accessing in real time that they have taken medications… and that the family members are there… was [a] challenging part*.” (Provider 3). These comments refer to pragmatic delivery within the TABI arm.

Patients described *social and emotional barriers* to adoption. In contrast, providers emphasized *selection and readiness* as key determinants of adoption success.

#### Implementation

Most patients and providers agreed that core treatment protocols, including drug history inquiries, informed consent, and structured follow-ups, were maintained under the TABI model. A patient recounted, *“The doctor explained if I used drugs while on medication*,* it could be dangerous*,* and they asked everything about my history.“*(Patient 1, 23 years).

However, *technology-related challenges* frequently disrupted smooth implementation. Many providers emphasized that *network connectivity issues* were persistent: *“In about 50 to 60% of cases*,* we couldn’t sustain a video call and had to switch to audio only.”* (Provider 3). Despite this, they adapted flexibly, rescheduling calls, simplifying instructions, and maintaining frequent communication. Many providers noted that smartphones were “easy to use” for most participants, but initial training and troubleshooting were necessary. Providers described *adherence-related difficulties* in a subset of patients, reflected in missed or delayed teleconsultations, inability to verify medication intake in real time, absence of family members during supervised dosing, delayed follow-up, and occasional relapse. These challenges were attributed not to a single factor but to a combination of low early motivation, limited family supervision, work and travel disruptions, and unstable phone or network connectivity.

Some providers reflected that the intervention taught them to better identify the patient profile best suited for telemedicine: *“Highly motivated patients with some family support did very well; for others*,* it was challenging.”* (Provider 1).

Patients sometimes preferred the tangible structure of in-person visits, especially when uncertain or under stress, stating plainly, “*No… it’s better to come here in person*” (Patient 6, 21 years). Providers likewise advocated periodic in-person reinforcement to stabilize care, “*once in a month follow-up in person visit will definitely go a long way*”, and noted that real-world distractions (patients “working, travelling, or driving”) often required rescheduling remote contacts. (Provider 3)

Both recognized *technology barriers*, but patients emphasized their *own lack of digital skills*, while providers emphasized *systemic connectivity barriers* and *technical quality of consultations*.

#### Maintenance

Both patients and providers emphasized that maintenance of treatment gains depended on structured follow-up, ongoing engagement, and psychosocial reinforcement. Patients valued the predictability of scheduled consultations: *“I have to come every 15 days depending on the doctor’s advice*,* and I follow their instructions.”* (Patient 4, 27 years).

Providers observed high retention rates and were “*pleasantly surprised*” by patients’ honesty and adherence: *“Some patients even returned unused medications during follow-ups*,* which was very encouraging.”* (Provider 1).

Challenges remained around *synchronizing teleconsultations and medication refills*. Providers recommended integrating pharmacy services more closely with telemedicine scheduling to prevent gaps in care.

Importantly, providers advocated for a *hybrid model* moving forward: *“Once-a-month in-person follow-up visits would strengthen the model further”* (Provider 3). They also emphasized the need to integrate *early motivational interventions* and *psychosocial support* to enhance long-term sustainability.

Scaling up TABI was seen as feasible and promising by providers, provided that logistical supports and patient training are systematically enhanced.

Patients focused on *logistical and social enablers* (scheduling, peer/family support) while providers focused on formalizing *hybrid models* (periodic physical reviews + ongoing teleconsultations) for sustained success.

Please see Table 1 (patients) and 2 (providers) for the detailed quotes, codes, sub-themes, and themes.

**Table 1:** Framework analysis of patients’/service users’ perspectives based on the reach-effectiveness-adaptation-implementation-maintenance domains

RE-AIM Domains / Themes	Sub Themes	Codes	Excerpts
Reach	Structural barriers	Travel and distance Waiting time at the clinic	“there’s no problem to come here for treatment, but they call me after a week. The issue is, I don’t get leave after a week because coming here takes time…”. “………….and then they don’t provide the medicine on time need to wait (Patient 6, 21 years)
		Financial Constraints	Just that it’s challenging sometimes to come for the medicines due to work. Catering work is unpredictable, but it’s essential for household expenses. Other than that, there’s no major issue. (Patient 8, 32 years)
			I was doing well in my career and my family was good, but my addiction led me to a bad path, damaging my image and weakening me financially. (Patient 10, 27 years)
	Logistical barriers/facilitators	Conflict between job and treatment Flexibility of clinics’ operation	Traveling isn’t an issue; the only thing is… Because of work, I sometimes get late. For example, if I don’t reach by 11, I manage to get in by 1 or 1:30, and I still get my medicine. (Patient 7, 34 years)
	Personal reasons	Addiction Awareness	Just that addiction should be avoided and everything should be fine. There should be focus on avoiding addiction. (Patient 12, 41 years)
		Awareness of Behavior	As my job is driving and when I drive the car, I used to feel sleepy, so to avoid sleep I take little opium, gradually it became my habit and my family members knew about it(Patient 14, 23 years)
		Counseling and Awareness	Someone had referred my mother to a boy in our area who knew about this place. He told us that there is a drug de-addiction center at PGI where they provide admission, medicines, and treatment. My mother and I came here, and after counseling, we learned about it. (Patient 8, 32 years)
		Intrinsic Motivation	The motivation came from here itself. (Patient 11, 27 years)
		Lack of Motivation	Earlier, I didn’t care about anything, not about eating or doing any work. (Patient 10, 27 years)
		Mental Health Awareness	There should be a dispensary provided by the government with the necessary medicines for mental health issues. (Patient 10, 27 years)
		Motivation to Quit	I felt I should quit because the medication relieved my pain, and I started feeling better. (Patient 4, 27 years)
Efficacy/ effectiveness	Psychological and Emotional Factors	Emotional Support	Yes, ma’am, I did receive support from my family. (Patient 7, 34 years)
		Motivation from Peers	My friend was taking medication here too. (Patient 12, 41 years)
		Adjustment to Routine	The problem is that I live alone. My mother passed away, I am divorced, and my baby also passed away. I live alone, so I have a job, and it’s a bit difficult to get leave to come here. (Patient 3, 35 years)
	Health System and Follow-Up Process	Clinical Support System	Yes, I have to come every 15 days or sometimes weekly, depending on the doctor’s advice. They schedule my visits, and I follow their instructions (Patient 4, 27 years)
		Challenges in Continuity	Just that it’s challenging sometimes to come for the medicines due to work. Catering work is unpredictable, but it’s essential for household expenses. Other than that, there’s no major issue. (Patient 8, 32 years)
		Medication side effects	When I was using drugs, my body was weakening. But even now, when I take medication, there is still some weakness in my body. (Patient 12, 41 years)
		Coping Difficulty	I would get angry, and if it was affecting me on one side, I couldn’t bear it. (Patient 5, 38 years)
		Early Challenges in Recovery	Yes, Initially, I faced. On the first day, there was some difficulty. (Patient 5, 38 years)
		Recovery Milestone	I am very happy, Ma’am. It feels like I have been given a new life after quitting addiction. I am very happy. (Patient 14, 23 years)
		Relapse Incident	Once I relapsed in a year, but then I restarted the medication. (Patient 5, 38 years)
Adoption	Family and social influence	Assistance by family	My father comes sometimes with me, my mother also comes sometimes. (Patient 13, 25 years)
		Role of Family Support	The doctor provided treatment, and my family also helped by guiding me. That’s why these changes happened. (Patient 5, 38 years)
		Motivation from peers	it was my friend who was saying that his treatment was working here, and he said that I should go there for treatment, and it would get better. Initially, I was not interested in the medicines. My friend explained it to me well, and I realized that I need to stop the addiction. (Patient 7, 34 years)
		Stigma within family	the way she explained everything to me… I was completely broken. At home, everyone called me an addict. No one talked to me with love. (Patient 3, 35 years)
		External Expectations	They also advised me to stay with my parents, and they asked why I haven’t quit yet despite the long time. They ask doctors why I haven’t stopped yet. (Patient 13, 25 years)
		Medication Discomfort	The reason is that after taking the medication, I don’t feel refreshed, and that’s why I take a gap. (Patient 6, 21 years)
		Physical Discomfort	I used to feel tired, didn’t want to get out of bed, didn’t want to do any work, and I also had body pain. (Patient 10, 27 years)
		Positive Evaluation of Doctors	They are okay. Yes, the doctors advise me well. (Patient 10, 27 years)
		Preference for In-person Visits	No, ma’am, it’s better to come here in person. (Patient 6, 21 years)
		Trust in Provider	I come here to get medicine for my addiction, I get my medication from Ha….et sir. (Patient 13, 25 years)
		Trust in Treatment	I do tell my friends and acquaintances to take the medicine; it’s a good treatment here. There’s no better treatment for drug addiction anywhere else. Whether private or government, the cooperation is there. (Patient 13, 25 years)
Implementation	Technology-Related Challenges	Lack of Technological Literacy	I don’t know. (Patient 14, 23 years)
		Drug History Inquiry	The doctor asked how long I’ve been using substances and how much I take. (Patient 12, 41 years)
		Informed Consent	The doctor asked how long I had been using drugs, what type of drugs I was using, and how I was using them. Then they explained the treatment process to me, and I signed the form. (Patient 2, 35 years)
		Treatment Explanation	the doctor explained that if I use drugs, I shouldn’t take the medicine. They also explained that if I use both, it could be harmful to my life. They also explained counseling to me, and I had a counseling session with the ma’am. She asked me everything in detail. (Patient 1, 23 years)
Maintenance	Family and Social Influence	Coordination with Family	My family brought me, especially my father. I’m not dependent in any way now. (Patient 13, 25 years)
		Peer Support Social Accountability	My friend was taking medication here too. (Patient 12, 41 years)
	Health System and Follow-Up Process	Follow-up Process	Yes, I have to come every 15 days or sometimes weekly, depending on the doctor’s advice. They schedule my visits, and I follow their instructions. (Patient 4, 27 years)
		External Recommendations	I have a friend who gets medicine from here. He told me about it. (Patient 10, 27 years)
		Follow-Up Needed	Nothing much. I was late today; they wrote today’s treatment and medicine. There was no entry on my card, just the medicine for today was written, and I need to come again tomorrow. (Patient 13, 25 years)
		Follow-up Communication	Yes, I get follow-up calls. I used to talk to my father as I didn’t have the number. (Patient 13, 25 years)
		Follow-up Scheduling Issues	I come from Sector 22. Sometimes the dates for the follow-up and the medicine collection overlap, and that causes problems. (Patient 10, 27 years)

### Cross-cutting themes across the RE-AIM domains

A cross-case, cross-domain synthesis revealed four transversal levers: family support, stigma, digital readiness/connectivity, and provider trust/rapport. Below, we describe how each theme operated across Reach, Adoption, Implementation, Effectiveness, and Maintenance, with illustrative evidence detailed in Tables 1 and 2.

**Table 2:** Framework analysis of treatment providers’ perspectives based on the reach-effectiveness-adaptation-implementation-maintenance domains

RE-AIM Domains/ Themes	Sub-themes	Codes	Excerpts
Reach	Accessibility and communication	Accessibility	“Like the accessibility was better it was like quite smooth and the outreach treatment services was very good, it was way more convenient to patient and also therapist in reaching the treatment related goals without much hassle and barrier so overall accessibility has been very good. It has been very promising” (Provider 2)
		Communication barriers	“there were some difficulties in engaging with the target population with this intervention like the phone use to be switched off and we used to call them or the number which was provided was invalid or the wrong number was given by the patients and few patients were reported the side effects excessive drowsiness some used to report dizziness etc. which was difficult to access in the real time through tele”. (Provider 3)
		Phone access Issue	“most of the patients they were like they were motivated for the tele medicine services but long term not long term the short term I think the first three days they were very poorly motivated. The time slot which was given they were not able to stick to time slot, when the call was made from our side either the phone is to be switched off or the attendants were not available. At times we used to video conferencing the video quality was so poor that it was not able to continue video, both audio plus video thing. Most of the time we disconnect the video part and continue the audio part only. So accessing the real time that they have taken medications in front of us plus accessing that the family members are there with them to supervise them medications was challenging part.” (Provider 3)
		Network connectivity barrier	what I remembered is the time given to them, when we will be following for the intervention or when will be accessing particular days were there 0 3 and 5 days some days we have decided. So during that time they were not available because their own commitments and that issue was second network issue that we faced. So, people coming from hilly area the network issue was the most important barrier that I faced. Other who had relapsed that day 2nd, 3rd day only of the therapy, so we understood that the motivation was not there that was one of the barrier. (Provider 1)
		Minor barriers to patient participation	“There were no major barriers as such minor issues such as network related issues which could hamper video calls but we could still connect on audio call only. Sometimes patients were in the middle of something for example they were working, travelling, or driving, etc during which was slightly difficult so we had to rescheduled the calls at times there were no major barriers I guess as such”. (Provider 4)
	Technology and Engagement	Lack of technology literacy	“like the major barriers is to like point out specially it is to be with a device related thing and also the patient unawareness like handling the devices or like being unaware with the tele-based programs how it goes exactly like the generally understanding amongst the patients is like reaching the hospital for the treatment centre in person so if the awareness is good and the kind of patient is educated and know how to handle the difficulties I think it can be a very good platform”. (Provider 2)
		Barriers to initial engagement and readiness	“so first of all it was quite new it was not an issue mostly literate people for technology was a barrier each and everything we had to explain each and everything to them in detail. The engagement was poor in them otherwise, once they got to know them the process they were engaging well. Apart from them, certain other factors like less motivation, lot of craving and not ready for quiting the substance, when they had included intervention, the intervention was for them since the start.” (Provider 1)
		Feasibility issues	“regarding accessibility patients were living far off those with physical issue those were like have some fractured or accident under the influence of the drug or they were not well due to some existing comorbidities disable to resisting to bed, they were enrolled mostly in that cases we enrolled through tele services. And also patient preference was also given that those who cannot come like after 3 days- 2 days because of the job issues cannot get repeatedly or the job was for accessibility issues there were feasibility issues, we enroll them through tele services”. (Provider 3)
	Convenience and benefits	Convenience	“Like the intervention I found to be very effective like in terms of it was more convenience for the patient and the outreach was very good. So overall it is a very good platform for a better like patient engagement” (Provider 2)
		Resource-efficient model	“So, it was effective because it bypasses the need for more frequent visits it was for the patients it was less time consuming and less resource consuming so we could engage better results also”. (Provider 4)
		Accessibility benefits	“Accessibility was different from in person in a way that it was more convenient for them and they were happy fact that took into consideration logistic issues like some of them were coming from different terrain and some of them have job problems they cannot simply come after just every day for ost starting initiation. It was quite different and they understood this fact that taking all this into consideration and I felt that all also meet them engage in the program well in in-person meet, and what had happen they sometimes they just doesn’t understand why we are calling them every day during phase so they sometimes come half-heartedly or that could be another stress and that stress could relapse also I feel that stress somewhere played good role while we were taking patients by telemedicine. (Provider 1)
Efficacy/ effectiveness	Treatment outcomes	Achieved treatment goals	“I think like effectiveness was quite good in terms of reaching the treatment related goals in overcoming the barriers that patient face in originally in hospitals and it can be a kind of overcame all these hassles, so overall the accessibility has been very good.” (Provider 2)
		Moderate effectiveness	the effectiveness of intervention in achieving its intended outcomes I would rate this 6 by 10 (Provider 3)
		Relapse incident	yes, like my second patient he was in some transport business and he has and used to travel a lot he was basically from Rajasthan so he would not call on a assigned timings given to him and the phone number which he had given us would be mostly switched off till 11 sometimes 12 pm and I had to have to call him multiple times during the working hours I used to call him 3 times so that I can supervise the medication on dose on his time on a given time or not and another issue difficulty which I faced was none of the time whenever I called there were no family members for supervising or whether he has taken medicine or not whether he has taken anything else other than medicine that part was missing though he used to say he is following everything but the thing is he used to consume extra tablets for that particular day and another thing is that he didn’t follow-up the 6thday which was lotted to him he came up after 20 days he has relapsed with opioids. (Provider 3)
		Patient satisfaction	I feel it was very effective because in fact treatment retention and patient satisfaction was better than the standard of care in this particular intervention. (Provider 4)
	Tele medicine effectiveness	Convenience of telemedicine	like in terms of treatment related outcomes I would say that there is no major differences between the telemedicine vs. in person settings, in those situation and circumstances like we discussed yet like tele based services and modalities has been like it was much more convenient and smoother like delivery the treatment. (Provider 2)
		Distance barriers to access	like in my experience I have during my residency days there were instances specially the patient would be from the far remote villages like Himachal and Punjab who would be founding like difficulties in turning because of the distance related issues. And also other like regions like harsh with the environment like any other factors and other challenges they face. (Provider 2)
Adoption		Consultant decision-making	there is no specific reason as such I think this thing was in decided by my consultants and I was allotted to what to do. (Provider 3)
		High telehealth acceptance	patients were accepting tele based interventions in fact more readily as compared to the inpatient visits for all the convenience which I just enumerate, they could save time, save the employability, money spending in the travel etc., so it was readily acceptable for the patients. (Provider 4)
Implementation	Technology and use	Network connectivity issues	one of the major challenges was I have mentioned earlier also the video part like most of the time 50 to 60% cases we were not able to make video call because if we start on video call audio quality becomes very poor. And the network used to be poor. Family members were also not there to supervise. (Provider 3)
		Ease of use	the tools were mainly smartphone which was very easy to use and it was quite convenient and the training was provided help us smoothly in implementing this intervention. (Provider 4)
		Adherence challenges	intervention was good enough but we have to, it worked in some patients and it didn’t work in some patients. And also that we eventually understood what kind of patients we should recruit for this sort of intervention for the one who are highly motivated and they have good family support coming from some, they have willingness to quit the motivation is in contemplation fraction phase somehow, they are not able to do they have actual logistic issue. Intervention turned out to be good but not for all the patients it was good. It might have acted in a wrong way because some of them didn’t turn up and it was some of the population, they took the ibuprofen and they didn’t come to the opd on a given day, so in that way it was problem but otherwise if we could choose the patient population precisely by keeping all this points into consideration, it can be a good intervention. (Provider 1)
Maintenance	Sustainability and Scaling	Scale-up recommendation	I think the future prospects regarding the scalability of this intervention model is very good, as the results are very promising. Results are positive. I think it should be implemented at a higher scale larger scale like more number of center deaddiction ya addiction related patients offered the benefits of mode of service, so I think it could be expanded at higher level. (Provider 2)
		Hybrid follow-up model recommendation	I think the teleservices is fine but I think once in a month follow-up in person visit will definitely go a long way. (Provider 3)
	Integration of Support Services	Need for early motivational intervention	what I feel personally is the first day or the first session they come to our OPD I think a regress motivational therapy should be done if we do enrol them on day 1 ofay fine we will start the tele service or whether we enrol in the patient services I think part is missing like the motivational part which is to be done on day 1 I don’t think it’s very effective I think we lack there I think things can be improved in this domain (Provider 3)
		Integrate psychosocial and infectious disease treatment	as I already mentioned psychosocial intervention and treatment for infectious diseases should be integrated into all this. (Provider 4)
		Follow-up adherence	so, I was quite apprehensive I feel that we are giving a lot of medications to them and we are following upto certain times they might not even follow-up but it I turned out to be wrong and I am happy about that the actually they came up for follow-up. Some of them also returned the medicine which we had given in excess in case they have a lot of craving. So it was quite surprising and happy experience for me that patients are being genuine. So it was nice overall experience. (Provider 1)

#### Family support

Family involvement functioned as an enabling condition throughout the cascade. For **Reach**, relatives frequently discovered services and facilitated first contact and logistics (e.g., accompanying patients, arranging appointments), helping patients overcome travel/time constraints. For **Adoption**, family members provided practical scaffolding—reminders, phone assistance, and early encouragement, that increased willingness to enroll and continue with TABI. During **Implementation**, the presence of a relative during induction days or scheduled calls often allowed real-time problem-solving (e.g., troubleshooting devices, supervising medication) when connectivity faltered. For **Effectiveness** (Efficacy/Effectiveness), patients linked emotional stability, adherence to routines, and early recovery milestones to family encouragement and accountability. Finally, for **Maintenance**, families sustained motivation between consultations and helped synchronize refills and follow-ups: one provider noted that supportive families were associated with “pleasantly surprising” honesty and return of unused medication, reinforcing long-term engagement (see Table 2). In short, family support acted less like a single-domain determinant and more like background infrastructure that amplified benefits of TABI across the continuum.

#### Stigma

Stigma, enacted within families and communities and internalized by patients, exerted multi-domain drag. At **Reach/Adoption**, being labeled “an addict” at home or fearing disclosure discouraged help-seeking, reduced readiness for change, and sometimes prompted preference for less visible contacts. During **Implementation**, stigma complicated synchronous supervision (e.g., reluctance to appear on video, arranging calls away from others), occasionally undermining safety checks and clinical observation. Providers’ reaction of being “pleasantly surprised” at the honesty of patients because they returned unused medications might also reflect structural stigma among care providers. Similarly, providers’ attribution of adherence challenges to *low intrinsic motivation* might also indicate a tendency to shift the blame to patients, inherently indicative of structural stigma. For **Effectiveness**, shame and negative affect interfered with coping, especially in early recovery or after lapses; patients described emotional volatility that made adherence harder even when medication helped. In **Maintenance**, persistent stigma threatened social reintegration and confidence during setbacks, indicating that anti-stigma and family-engagement strategies are not ancillary but central to sustaining telemedicine-enabled OAMT.

#### Digital readiness and connectivity

Device availability, phone number stability, digital literacy, and network quality jointly acted as a structural gatekeeper that touched every domain. For **Reach**, lack of a reliable device/number or low digital literacy limited who could engage quickly and consistently. In **Implementation**, providers frequently downgraded video to audio, rescheduled calls, or layered extra instructions; in some weeks, half or more of scheduled video contacts could not be maintained due to bandwidth constraints, especially for patients in hilly/rural areas (Table 2). These disruptions spilled into **Effectiveness**, where missed observation and weaker non-verbal assessment increased uncertainty around medication intake and risk behaviors, and into **Maintenance**, where poor synchronization between teleconsultation timing and pharmacy refills produced avoidable gaps. Patients’ self-reported digital inexperience (“I don’t know”) contrasted with providers’ system-level view (valid/invalid numbers, switched-off phones, weak video), underscoring that “digital access” entails both individual capability and infrastructural reliability. Targeted onboarding and minimal-friction workflows appear pivotal for equitable scale-up.

#### Provider trust and rapport

A strong therapeutic relationship emerged as the relational engine linking initial acceptance to sustained engagement. For **Adoption**, trust in clinicians—manifest as confidence in explanations, feeling “advised well,” and naming specific clinicians as anchors—helped patients choose and stick with TABI. During **Implementation**, rapport buffered the limitations of remote care; when video failed or sessions were brief, prior relational equity sustained cooperation and disclosure. In **Effectiveness**, regular, empathic check-ins supported motivation, emotion regulation, and adherence, aligning patients’ narratives of “new life” and functional improvement with clinician-rated progress. For **Maintenance**, relational continuity fostered honesty about lapses and safe return to care, while providers advocated periodic in-person “relational boosts” (e.g., monthly visits) to reinforce alliance, enable direct observation, and recalibrate plans.

### Difference and overlap between patients and providers

Patients and providers agreed that TABI improved convenience, but their emphasis differed. Patients highlighted the practical work of keeping care on track, saying “*I don’t get leave after a week… coming here takes time*,*” “it’s a bit difficult to get leave to come here.”* They also described the motivational lift of progress: “I have been given a new life after quitting addiction.” Providers focused on clinical feasibility, describing contact and bandwidth problems: “the phone was switched off, numbers were invalid, or the video quality was so poor that we had to disconnect the video and continue with audio,” and early variability in readiness: “the first three days they were very poorly motivated.” They also emphasized selecting the right profile for success: “highly motivated patients with good family support did very well,” while still acknowledging patient-perceived value: “treatment retention and patient satisfaction was better.” Taken together, the model works best when patients lived logistics such as leave from work, travel, and refill, teleconsultation timing are made manageable, and the service secures clinical conditions such as stable contact, adequate bandwidth, and early motivational support.

### Mechanism of effectiveness and implementation pathway of TABI

This conceptual model illustrates how TABI operates within a layered psychosocial and structural environment to influence patient engagement, treatment experience, and long-term outcomes. Informed by the RE-AIM framework and grounded in qualitative findings, the model traces the patient journey from initial engagement to sustained recovery, accounting for both technological and human factors.

Importantly, this model was not intended to compare the TABI and in-person arms of the trial. Rather, it served as an analytic tool to contextualize the RE-AIM findings within a broader continuum of care and to inform recommendations for improving the acceptability, feasibility, and sustainability of the TABI approach in resource-constrained settings.

#### Community context

The model begins with the immediate social context most relevant to TABI uptake and engagement, particularly family involvement, peer influence, and stigma within close social networks. In our data, family members often facilitated treatment access by identifying services, accompanying patients, or helping them connect during follow-up calls, while peers sometimes encouraged treatment seeking by sharing positive experiences. At the same time, stigma within the family could undermine readiness and emotional confidence. In this sense, TABI did not remove stigma as a barrier, but for some participants it appeared to lessen the practical and social burden of seeking care by allowing with less repeated public travel to the clinic.

#### Initial engagement

Individuals transition into *initial engagement*, where the decision to initiate care is determined by a confluence of factors such as *accessibility of provider*, *travel barriers*, *financial constraints*, and *information-seeking behavior*. Participants often described the *approachability of providers* and the ease of connecting with services via phone as enablers of engagement, particularly for those who faced challenges with transportation or daily wage work. Conversely, others highlighted that limited outreach and a lack of clarity around service options could inhibit timely access. The role of *motivation to participate*, often shaped by previous negative experiences or external encouragement, also featured prominently in this phase.

#### Mode of delivery and contact modality

In the parent trial, allocation to TABI vs. in-person induction was randomized. The model summarizes experiences within TABI, noting perceived advantages (flexibility) and constraints (digital literacy, connectivity). Some participants also described valuing the tangible structure of in-person contacts during uncertainty.

#### Treatment delivery and implementation

Treatment fidelity and patient satisfaction were shaped by the quality of *treatment delivery and implementation*. Participants emphasized the importance of clear *treatment explanation*, timely *informed consent*, and comprehensive *drug history inquiry*. Consistent *provider rapport* emerged as a key driver of trust, and *emotional support* from providers was described as critical for maintaining motivation during early recovery. Core clinical protocols (history, consent, counselling, structured follow-up) were preserved during in-person induction and reinforced in tele-follow-ups within TABI; connectivity sometimes shortened or altered remote interactions. Notably, *dependency on staff* and the perceived *adequacy of treatment duration* influenced how supported participants felt during the induction phase.

Providers acknowledged that the inability to consistently monitor non-verbal cues during remote interactions posed a challenge for comprehensive clinical assessment. Adaptations, such as switching between video and audio modalities or supplementing virtual check-ins with in-person reviews, when necessary, were commonly employed to maintain implementation fidelity.

Patients’ perceptions of support were also influenced by their dependency on provider communication and their subjective assessment of whether the treatment duration was adequate. Those who felt that follow-ups were too brief or insufficiently personalized occasionally expressed concerns about feeling less supported, highlighting the importance of maintaining relational depth even within a telemedicine framework.

#### Treatment outcomes and experience

Patient experience during treatment reflected a diverse set of outcomes. Many described *perceived recovery*, citing improvements in *daily functioning*, better family interactions, and enhanced emotional regulation. Participants also discussed *engagement fluctuation*, often related to external stressors or lapses in connectivity, and acknowledged *adjustment to routine* as a meaningful marker of progress. Overall, the majority expressed *treatment satisfaction*, recognizing the role of the structured intervention in facilitating behavior change.

#### Follow-up and maintenance

**Fig. 1:**
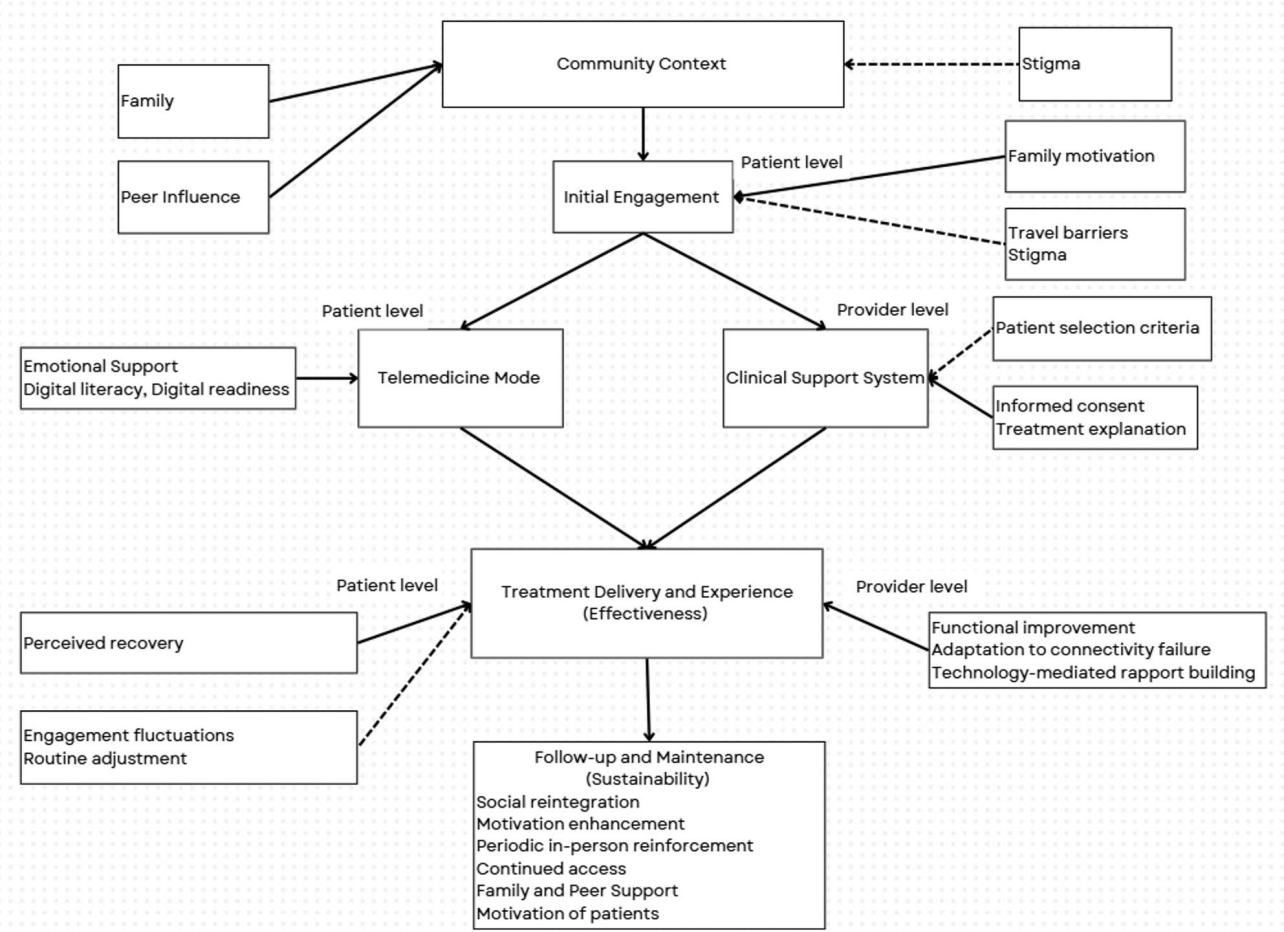
Mechanism of Implementation and Effectiveness of Telemedicine-Assisted Buprenorphine Induction (TABI). The model depicts how family support, stigma, and peer influence shape initial engagement in TABI. Treatment delivery via telemedicine or in-person care leads to recovery milestones through emotional support, clinical explanation, and provider rapport. Sustainability is supported by continued access, family and peer reinforcement, and social reintegration, highlighting the dynamic interplay of individual, technological, and systemic factors. Bold arrows (→) represent facilitators; dotted arrows (- - >) represent barriers. Themes are derived from participant interviews and reflect patient- and provider-level influences on telemedicine-based care

The final stage of the pathway addresses *follow-up and maintenance*, focusing on the sustainability of treatment benefits. Many participants highlighted the importance of *continued access* to providers and routine follow-up, particularly for managing vulnerability to relapse. Ongoing *family and peer support* was critical for reinforcing recovery goals, especially in the face of persistent stigma or socioeconomic stress. Long-term *motivation* was often grounded in a desire for *social reintegration*, financial independence, or improved familial roles. Yet, participants also expressed concerns about the stability of recovery in the absence of structured check-ins or when facing personal crises. A few articulated the need for system-level reforms to extend care beyond induction, ensuring that the intervention model could evolve into a sustainable continuum. Providers echoed the importance of structured follow-up but emphasized additional system-level strategies for improving maintenance outcomes. They advocated for integrating periodic in-person reinforcement, suggesting that monthly physical visits could strengthen therapeutic alliances, allow direct clinical observation, and mitigate the limitations of purely remote care. Providers also proposed embedding early motivational enhancement strategies during telemedicine sessions to fortify patient commitment during vulnerable phases (Fig. 1).

## Discussion

This qualitative process evaluation embedded within a randomized controlled trial provides in-depth insights into the delivery and experience of TABI in a resource-constrained setting in India. By applying the RE-AIM framework, we explored how TABI expanded access, improved perceived clinical effectiveness, and was perceived by patients and providers. We also identified the practical challenges that shaped adoption, implementation, and maintenance.

Our findings suggest that TABI successfully lowered structural barriers to treatment initiation, especially travel-related burdens and scheduling conflicts. These findings mirror observations from high-income settings where telemedicine-delivered medications for opioid use disorder (TMOUD) have reduced logistical barriers and expanded reach, particularly during the COVID-19 pandemic [16, 17]. However, consistent with prior literature, we also identified residual barriers, including limited digital literacy and inconsistent network connectivity, that may differentially affect marginalized populations [18].

Participants largely perceived TABI as clinically effective, reporting reductions in opioid use, improved daily functioning, and enhanced emotional stability. Similar positive treatment outcomes have been reported in telemedicine-based MOUD programs globally [19, 20], reinforcing the potential of telehealth to maintain core clinical benefits even when delivered remotely. Nevertheless, the emergence of coping difficulties, emotional fluctuation, and occasional relapse incidents in our study highlights the need for ongoing psychosocial support beyond medication management, an aspect emphasized in prior work on holistic OUD care models [21].

Among participants already engaged in treatment, acceptance of TABI appeared to be shaped by practical convenience and immediate social context. Trust in providers, family encouragement, and peer influence supported engagement with telemedicine follow-up, while stigma within families could still affect comfort and confidence during treatment. These findings echo earlier research indicating that stigma operates as a persistent barrier to treatment uptake for in-person and telehealth-based treatment for OUD, even when logistical access is improved [22, 23]. Interventions to systematically address stigma within telemedicine-based OUD models warrant further exploration.

Implementation fidelity was largely maintained, with treatment explanation, informed consent, and clinical assessments consistently reported by participants. These findings suggest that clear communication and person-centered clinical engagement remained important during telemedicine delivery [24], even though key treatment decisions had largely been made earlier during diagnosis, eligibility assessment, and enrollment. However, technological challenges, including low digital literacy and network instability - intermittently disrupted the delivery of care. These observations are consistent with concerns raised in other telemedicine evaluations from the global north [19, 25] and suggest that successful scaling of TABI will require supplementary digital support strategies, including patient training and infrastructure improvements.

Sustainability of the TABI model was seen as contingent on structured follow-up, ongoing provider contact, and family or peer reinforcement. Participants emphasized the importance of maintaining relational continuity even through remote platforms, highlighting the relational underpinnings of effective addiction care [26]. Fostering therapeutic alliance needs a systemic integration of training of healthcare providers and supervisors, and its conscious and deliberate application in the treatment settings [27]. Challenges with synchronizing medication dispensing and teleconsultations underscored the need for stronger integration of pharmacy and telemedicine logistics. Finally, patients’ accounts of improvement were not limited to opioid use reduction alone, but also included recovery-oriented experiences such as regaining routine and improved functioning. However, these outcomes were not commonly reported in clinical trials, which were more inclined to report abstinence and treatment retention [28]. Recent consensus initiatives have proposed a core outcome set for opioid use disorder treatment research, i.e., a minimum, standardized set of outcomes that trials should report, with North American and European panels independently recommending domains beyond abstinence and retention to include patient-reported functioning, quality of life, and mental health [29, 30]. The European consensus that included people with lived experience had reported more person-centered outcomes (quality of life, social and individual functioning, and mental health-related outcomes) than the North American consensus, which was primarily represented by researchers, service providers, and administrators. Our study suggests that the voices of people with OUD, with lived and living experience, and from the global south, must be heard for deciding on the core set of outcomes.

An important insight from this process evaluation was the relative difference in emphasis between patients and providers regarding their experiences with TABI. Patients primarily focused on their lived realities, emphasizing convenience, emotional coping, peer and family influences, and logistical challenges such as work conflicts and financial constraints. In contrast, providers were more concerned with systemic and procedural factors, such as maintaining treatment fidelity, technology infrastructure, and ensuring clinical appropriateness of telemedicine for certain patient profiles. This divergence mirrors findings from broader research on telemedicine-delivered OUD care. Across multiple studies, patients described telemedicine as enhancing privacy, convenience, and reducing stigma, often feeling more comfortable and empowered during virtual consultations [31, 32]. However, providers expressed concerns about building therapeutic rapport remotely, difficulties in assessing non-verbal cues, supervising medication adherence, and ensuring accountability [33, 34]. Furthermore, providers highlighted the necessity of patient selection, favoring telehealth for stable or lower-acuity individuals, while patients rarely mentioned this stratification themselves [31]. These subtle tensions in expectations and experiences emphasize the importance of co-designing telemedicine models that are both patient-centered and clinically robust. Future iterations of TABI could incorporate additional digital literacy support, enhance motivational interviewing approaches during teleconsultations, and formalize hybrid models with periodic in-person visits to align patient and provider priorities [32, 33].

A major strength of this study is the use of the RE-AIM framework to systematically assess patient and provider experiences across the treatment cascade. Additionally, by capturing perspectives from both stakeholders and using saturation-driven sampling, the findings offer comprehensive insights into implementation dynamics. At the same time, relying on a single a priori framework to guide data collection and analysis can over-structure interpretation, risking a reductionist account of complex, context-dependent interventions and behaviors. The sample was limited to male patients, reflecting the gender distribution of the trial cohort, and findings directly did not capture the experiences of women or other marginalized groups. Although two women were enrolled in the parent trial, they could not be reached for interview, limiting inclusion of gendered perspectives, particularly around stigma, and our interpretations should be read with this constraint in mind. This qualitative evaluation was single-site, and all provider interviews came from the same tertiary-care facility, which may limit transferability of implementation insights to settings with different staffing, digital infrastructure, pharmacy workflows, or patient mix. Because the sample comprised clinic-seeking individuals randomized within a tertiary-care trial, our Adoption findings pertain to modality uptake among enrolled patients rather than population-level uptake in the community.

These findings can be operationalized as a site-readiness checklist (family engagement, stigma mitigation, digital onboarding, refill synchronization, hybrid follow-ups) to support TABI roll-out in other settings. Multi-site hybrid effectiveness-implementation studies should assess generalizability, cost, and equity impacts. Future research should explore adaptations of TABI that address the digital divide, integrate stigma-reduction strategies, and embed structured psychosocial interventions alongside medication delivery. Policymakers and program designers seeking to expand access to OAMT in low- and middle-income countries may consider telemedicine models like TABI as a scalable solution, provided that systemic barriers are proactively addressed.

TABI was experienced as acceptable and feasible, with perceived advantages in practical access and continuity when travel, distance, or work-leave constraints limited in-person care. Cross-cutting factors, family scaffolding, stigma, digital readiness/connectivity, and therapeutic alliance, shaped reach, uptake, implementation fidelity, and maintenance.

## Supplementary Information

Below is the link to the electronic supplementary material.

Supplementary Material 1

Supplementary Material 2

## Data Availability

No datasets were generated or analysed during the current study.
