# Supplementary material for: From access to sustainability: understanding telemedicine-based buprenorphine induction through a RE-AIM lens
Source: Addict Sci Clin Pract. 2026 May 26;21:55. doi: 10.1186/s13722-026-00670-6 (PMC13403578; doi:10.1186/s13722-026-00670-6)
Supplement: Supplementary file 1 — Supplementary Material 1 [file 13722_2026_670_MOESM1_ESM.docx]

Supplementary 1: In-depth Interview Guide for Patients (RE-AIM)

**Reach**

1. How did you learn about this treatment program?

2. What motivated you to participate in this specific mode of treatment (telemedicine or

in-person)?

3. Do you feel that the treatment was accessible to you? Why or why not?

**Efficacy/Effectiveness** 4. Can you describe any changes you’ve noticed in your health or

behavior since starting the treatment?

5. What aspects of the treatment do you think were most helpful in making these

changes?

6. Were there any obstacles or challenges you faced during the treatment that affected

your recovery?

**Adoption** 7. How did you find the environment where you received treatment (online or

physical location)? Was it welcoming and suitable for your needs?

8. Was there anything that could have been improved in how the treatment was

offered?

**Implementation** 9. How closely did your treatment follow what was explained to you at the

beginning? Were there any deviations?

10. Can you describe a typical session of your treatment? What activities or interactions

did it involve?

11. How did the healthcare professionals engage with you during the treatment? Were

they supportive and attentive to your needs?

**Maintenance** 12. Do you feel the benefits you gained from the treatment are sustainable

over the long term?

13. What kind of follow-up or support do you think is necessary to maintain the benefits

from the treatment?

14. Would you recommend this treatment method to others with similar conditions? Why

or why not?

**General Questions** 15. Overall, how satisfied are you with the treatment you received?

16. Is there anything else about your experience that you would like to share that we

haven’t already discussed?
