# Supplementary material for: From access to sustainability: understanding telemedicine-based buprenorphine induction through a RE-AIM lens
Source: Addict Sci Clin Pract. 2026 May 26;21:55. doi: 10.1186/s13722-026-00670-6 (PMC13403578; doi:10.1186/s13722-026-00670-6)
Supplement: Supplementary file 2 — Supplementary Material 2 [file 13722_2026_670_MOESM2_ESM.docx]

Supplementary 2: In-depth Interview Guide for Treatment Providers (RE-AIM based)

**Reach**

1. From your perspective, how effectively were we able to engage the target population

with this intervention?

2. What were the major barriers to patient participation that you observed?

3. How did patient accessibility to the program differ between telemedicine and in-

person modalities?

**Efficacy/Effectiveness** 4. In your view, how effective was the intervention in achieving its

intended outcomes?

5. What differences, if any, did you notice in patient outcomes between telemedicine

and in-person settings?

6. Can you share any specific patient success stories or notable failures?

**Adoption** 7. What were your initial thoughts about delivering the intervention via

telemedicine versus in-person?

8. What factors influenced your decision to participate as a provider in this trial?

9. Have you observed any changes in the organizational support for each mode of

delivery over time?

**Implementation** 10. How well do you think the intervention was implemented according to

the protocol? Were there any deviations?

11. What challenges did you face in implementing the interventions as planned?

12. How did the tools and resources provided (e.g., training, technology) support or

hinder the treatment delivery?

**Maintenance** 13. Based on your experience, do you believe the benefits of the intervention

are sustainable over the long term for patients?

14. What are your thoughts on the scalability of this intervention model?

15. What steps would you recommend for improving long-term patient support post-

intervention?

**General Questions** 16. How satisfied are you with the overall process of this clinical trial?

17. What improvements would you suggest for future iterations of this treatment model?

18. Are there any other insights or observations you would like to share that we haven’t

covered?
